# Human Gait Recognition Based on Multiple Feature Combination and Parameter Optimization Algorithms

**DOI:** 10.1155/2021/6693206

**Published:** 2021-02-27

**Authors:** Farong Gao, Taixing Tian, Ting Yao, Qizhong Zhang

**Affiliations:** School of Artificial Intelligence, Hangzhou Dianzi University, Hangzhou 310018, China

## Abstract

Accuracy is a key index of human gait recognition. In this paper, we propose an improved gait recognition algorithm, which combines multiple feature combination and artificial bee colony for optimizing the support vector machine (ABC-SVM). Firstly, considering the complexity characteristics of surface electromyography (sEMG) signals, four types of features are extracted from the denoised sEMG signals, including the time-domain features of integral of absolute value (IAV), variance (VAR), and number of zero-crossing (ZC) points, frequency-domain features of mean power frequency (MPF) and median frequency (MF), and wavelet features and fuzzy entropy features. Secondly, the classifiers of SVM, linear discriminant analysis (LDA), and extreme learning machine (ELM) are employed to recognize the gait with obtained features, including singe-class features, multiple combination features, and optimized features of dimension reduction by principal component analysis (PCA). Thirdly, the penalty coefficient and kernel function parameter of the SVM classifier are optimized by the ABC algorithm, and the influence of different features and classifiers on the recognition results is studied. Finally, the feature samples selected to construct the SVM classifier are trained and recognized. Results show that the classification performance of the ABC-SVM classifier is significantly better than that of the nonoptimized SVM classifier, and the average recognition rate is increased by 3.18%. In addition, the combined feature samples (time-domain, frequency-domain, wavelet, and fuzzy entropy features) not only improve the gait classification accuracy but also enhance the recognition stability.

## 1. Introduction

Gait refers to the movement and balance of the human body when walking upright, which is the basic movement mode of the lower limbs [1, 2]. Under the control of the nervous system, gait is completed by the joint action of muscles, joints, and bones. The gait phase in a walking cycle can be divided into support and swing phases [3]. Surface electromyography (sEMG) is a kind of weak bioelectrical signal produced with muscle contraction and is related to nerve function stimulation and muscle and movement states [4]. Gait pattern recognition technology based on sEMG signals has significant research value in the fields of intelligent prosthetic control and assisted rehabilitation [5].

Feature extraction is an important part of EMG signal analysis. The features are generally used to analyze sEMG signals, including time-domain (TD) features, frequency-domain (FD) features, wavelet features, and nonlinear features [6]. The time-domain features treat the EMG signals as a function of time and analyze the signal to obtain statistical features. Feature extraction is commonly used as the feature sample of the EMG signals because of its mature technology and relatively simple extraction. Time-domain features include integral of absolute value (IAV), variance (VAR), and number of zero crossing (ZC) [7, 8]. Li et al. [9] proposed gait recognition based on sEMG with different individuals and sample sizes. However, sEMG causes severe mutations in the time-domain features and has relatively poor stability due to its susceptibility to fatigue and other factors [10]. Compared with time-domain analysis, frequency-domain analysis solves the problem of signal instability in time-domain analysis by transforming the EMG signals into frequency or power spectra through Fourier transform, mainly including average power and median frequency (MF) [11, 12]. Pancholi and Joshi [13] proposed the extraction of time- and frequency-domain features of the EMG signals and used different classifiers for classification and recognition of different muscles. Analysis of sEMG through wavelet transform has shown remarkable vitality in recent years. Compared with the Fourier transform, the wavelet transform is a local transformation in space and frequency domains. The wavelet transform performs multiple scale refinement and decomposition of the signal through scaling and translation, thus effectively extracting information from the signal [14]. Ntsama et al. [15] compared the two methods of discrete cosine transform and discrete wavelet transformation and found that the effect of discrete wavelet transformation is effective. Duan et al. [16] used wavelet transform to utilize the maximum absolute value of wavelet coefficients as feature vectors to recognize hand movements and obtain good recognition results. Fuzzy entropy (FuEn) can perform nonlinear analysis on EMG signals and quantify the complexity by calculating the regularity of time series [6]. Chen et al. [17] compared approximate, sample, and fuzzy entropies for knee osteoarthritis research and found that FuEn has the best performance. Chen et al. [18] used FuEn as the feature samples to study the recognition of human lower limb gait movements. An increase in the feature dimension will also increase the training time; therefore, the features should be reduced before recognition. A traditional feature contraction method is conducted through principal component analysis (PCA) [19]. The combination of PCA and neural network can be used for finger motion recognition and posture control of the hand movements [20, 21]. In addition, several new algorithms have been proposed recently to improve the search quality, scalability, efficiency, and stability, as well as the recognition rate [22–24].

With the advancement of measurement technology and recognition algorithms, support vector machine (SVM), linear discriminant analysis (LDA), and extreme learning machine (ELM) are widely utilized for pattern recognition [25–28]. Wang et al. [29] identified multiple gait patterns and compared the classification performance of SVM and neural networks. Their results demonstrate that the classification performance of SVM is the best. Huang et al. [30, 31] proposed the ELM algorithm based on the feedforward neural network with a single hidden layer, in which the weights and biases are randomly assigned following the distribution of training samples. Tian et al. [32] presented the ELM to recognize the gait to achieve effective control of the prosthesis. Compared with the neural network, the results show that ELM has a fast recognition speed and high accuracy. On the basis of regularized extreme learning machine (RELM) [33], Deng et al. [34] proposed a continuous motion estimation algorithm with the combination of PCA and RELM. Bellingegni et al. [35] utilized the EMG signals to compare the classification effects of four different classifiers, i.e., nonlinear logistics regression (NLR), multilayer perceptron (MLP), SVM, and LDA. Their results show that the classification performance of SVM is better than the three other methods. Gu and Liu [36] used an improved SVM to identify six types of arm motion patterns. Lu et al. [37] studied the control of manipulators through sEMG signals and SVM classification model. Wu et al. [38] employed SVM for classification and recognition of the upper limb with single-channel sEMG signals.

The key problem of SVM is how to determine its penalty coefficient and kernel function parameter. The optimization algorithm is primarily adopted to solve the problem of parameter optimization in practical operation, and the optimized SVM is applied to classification and recognition. Zhang et al. [39] used the ant colony optimization (ACO) to optimize SVM parameters to obtain good results. The ant colony algorithm has satisfactory search capability, but its convergence speed becomes slow. Deng et al. [40] proposed an effective improved co-evolution ant colony optimization algorithm with multiple strategies and its application. Wang et al. [41] adopted particle swarm optimization (PSO) to optimize the parameters of SVM to achieve wind power prediction. PSO has the advantages of fast search speed and simple structure but lacks local optimization capability [42]. Li et al. [43] proposed the genetic algorithm (GA) to optimize the parameters of SVM for gait recognition. However, GA coding and decoding are complicated, and the parameters involved in the three operators are generally determined by experience. Kuo et al. [44] presented the artificial bee colony (ABC) to implement feature selection and SVM parameter optimization. Compared with GA and PSO algorithms, the ABC algorithm was fit to optimize SVM (ABC-SVM) to improve the classification accuracy and complexity simultaneously. Huang et al. [45] optimized the SVM parameters through ABC and realized the analysis of dissolved gases. Li et al. [46] compared the four algorithms of ABC, GA, PSO, and ACO and found that the ABC algorithm has a simpler operation, fewer parameters, and stronger search capability than other algorithms.

The main contributions of this study can be outlined as follows: (1) a multiple feature combination for sample selection is proposed and (2) an ABC-SVM parameter optimization algorithm for gait classification is improved, which can be used to increase the recognition accuracy and improve the classification performance.

The remainder of this paper is organized as follows. The principles and methods, including four types of feature extraction and four classes of classification recognition algorithms, are introduced in Section 2. The results of gait recognition based on multiple feature combination and parameter optimization algorithms are discussed in Section 3. Finally, the conclusion is given in Section 4.

## 2. Principles and Methods

### 2.1. Feature Extraction of sEMG Signals

#### 2.1.1. Time-Domain Feature

The wavelet modulus maximum method is initially used for the collected sEMG signals in the experiment for noise elimination [47], and the time-domain features with large separation, good robustness, and low operation dimension, such as integral of absolute value, variance, and number of ZC, are extracted [6].

*Integral of Absolute Value (IAV)*. The sEMG signals can be approximately regarded as a random signal with a mean value of zero, and the direct use of the mean value as the feature value of the sEMG signals cannot reflect the characteristic information. Therefore, the IAV is used as the time-domain feature to indicate the changes in the sEMG signal amplitude in the time domain, which is expressed as follows:

(1) $$\begin{array}{c} \text{IAV}=\frac{1}{N}\sum_{k=1}^{N}\left|x_{k}\right|, \end{array}$$

where *x*_*k*_ represents the sEMG signal value at *k* point, that is, *k*=1,2,…, *N*, and *N* represents the characteristic window size of the sEMG signals.

*Variance (VAR)*. Variance is used as a time-domain feature to measure the power of the sEMG signals, that is,

(2) $$\begin{array}{c} \text{VAR}=\frac{1}{N-1}\sum_{k=1}^{N}x_{k}^{2}. \end{array}$$

*Zero Crossing (ZC)*. The sEMG signal is derived from the electrical pulse sent by the nervous system, and its intensity is related to the frequency of the electrical pulse. The number of ZC refers to the number of times that the sEMG signal waveform passes through the zero point, that is,

(3) $$\begin{array}{c} \text{ZC}=\sum_{i=1}^{N-1}\mathrm{sgn}\left(-x_{k}x_{k+1}\right), \end{array}$$

where *k*=1,2,…, *N* − 1, $\mathrm{sgn}\left(x\right)=\left\{\begin{array}{cc} 1, & x>0,\\ 0, & \text{other}, \end{array}\right.$. In addition, setting a threshold can effectively avoid the signal cross counting due to noise.

#### 2.1.2. Frequency-Domain Feature

Extracted time-domain features are unstable because the EMG signals are weak signals and easily affected by noise. The frequency-domain features are converted into the midfrequency and power spectra of the frequency domain through fast Fourier transform, which can be directly observed from the frequency-domain distribution of the sEMG signals [11]. The power spectrum *P*(*f*) of the sEMG signals is calculated as follows:

(4) $$\begin{array}{c} P_{x}\left(e^{jw}\right)=\underset{M⟶\infty }{\lim}E\left\{\frac{1}{2M+1}{\left|\sum_{n=-M}^{M}e^{-jwn}\right|}^{2}\right\}. \end{array}$$

Equation (4) is applied to estimate the power spectrum of the sEMG signals of finite length and calculate the mean power frequency (MPF) and median frequency (MF).

(5) $$\begin{array}{c} \text{MPF}=f_{m}=\frac{\int_{0}^{+\infty }fP\left(f\right)\text{d}f}{\int_{0}^{+\infty }P\left(f\right)\text{d}f},\\ \int_{0}^{\text{MF}}P\left(f\right)\text{d}f=\int_{\text{MF}}^{+\infty }P\left(f\right)\text{d}f=\frac{1}{2}\int_{0}^{+\infty }P\left(f\right)\text{d}f, \end{array}$$

where *P*(*f*) is the power spectral density function of the sEMG signals and MFis the required median frequency.

#### 2.1.3. Wavelet Feature

Wavelet decomposition (WD) is an effective tool for time-frequency analysis and processing of sEMG signals. Mallat algorithm is the mainstream method of wavelet decomposition [16]. Suppose the original signal is *x*, then the wavelet decomposition formula is as follows:

(6) $$\begin{array}{c} \left.\begin{array}{c} s_{i+1}=Hs_{i},\\ d_{i+1}=Gd_{i}, \end{array}\right\} \end{array}$$

where *i*=0,1,…, *P*, *P* represents the maximum number of decomposition layers, *s*_*i*_ represents the low-frequency component with a resolution of 2^*i*^, namely, approximate component, *d*_*i*_ represents the high-frequency component with a resolution of 2^*i*^, namely, the detail component, and *H* and *G* represent low- and high-pass filters, respectively, as expressed by the matrix.

The decomposed mean and singular values are extracted as wavelet features, which are presented as follows:

(7) $$\begin{array}{c} J=\frac{1}{M}\sum_{i=1}^{M}q_{i},\\ A=u*Q*v^{\prime }, \end{array}$$

where *J* is the mean of the approximate component and detail component, *i*=1,2,…, *M*, *M* is the size of the feature window, and *q*_*i*_ is the approximate component and detail component signal value at point *i*. *A* represents a one-dimensional matrix of data in the window, *u* and *v* are the singular vectors of the matrix of *A*, and *Q* is the singular value matrix.

#### 2.1.4. Fuzzy Entropy Feature

Fuzzy entropy is a measure of sequence complexity and can be used to describe the complexity of a signal. The complexity of the sEMG signals is analyzed in this study by extracting fuzzy entropy [18]. The fuzzy entropy value (FuEn) is

(8) $$\begin{array}{c} \text{FuEn}\left(n,N,r\right)=\ln\;\Phi^{n}\left(n,N,r\right)-\ln\;\Phi^{n+1}\left(n,N,r\right), \end{array}$$

where Φ represents the similarity function, *N* is the time series dimension, *n*(*n* ≤ *N* − 2) is the phase space dimension, and *r* is the similarity tolerance.

### 2.2. Classification Recognition Algorithms

#### 2.2.1. Support Vector Machine (SVM)

SVM is a classification technique used for pattern classification and nonlinear regression [48]. The idea is to transform the vector map into a high-dimensional space and find the nonlinear relationship between the input quantity **z** and the output quantity *y* in the high-dimensional space. That is, the input space is converted to a high-dimensional space by setting a suitable kernel function, and the optimum linearity classification facet is obtained in the high-dimensional space. The decision function of the output classification *f*(**z**) can be defined as

(9) $$\begin{array}{c} f\left(z\right)=\sum_{i=1}^{m}\alpha_{i}y_{i}K\left(z,z_{i}\right)+b,\;0\leq \alpha_{i}\leq C, \end{array}$$

where **z** represents input feature vectors, *a*_*i*_ represents the Lagrange multiplier corresponding to each training sample, *C* represents the penalty coefficient, *b* represents bias, and *K*(**z**, **z**_*i*_) represents the kernel function, namely,

(10) $$\begin{array}{c} K\left(z,z_{i}\right)=\exp\left(-\frac{{\left\|z_{i}-z\right\|}^{2}}{g^{2}}\right). \end{array}$$

The kernel function in SVM is obtained. The kernel function parameter *g* and penalty coefficient *C*, respectively, determine the accuracy and generalization capability of the algorithm.

#### 2.2.2. Linear Discriminant Analysis (LDA)

LDA projects high-dimensional sample feature data into the best classification vector space [35]. The distance between different classes in the classification vector space after projection is the largest, and that within the same class is the smallest, so the sample pattern has the best classification effect in the projected subspace. The LDA criterion aims to maximize the ratio of the divergence between the classes of the sample to the divergence within the classes as shown in the following equation:

(11) $$\begin{array}{c} J\left(W\right)=\underset{w}{\arg\max}\frac{\left|S_{b}\right|}{\left|S_{w}\right|}=\underset{w}{\arg\max}\left|\frac{W^{T}S_{b}W}{W^{T}S_{w}W}\right|, \end{array}$$

where *S*_*b*_ and *S*_*w*_ represent the interclass and intraclass dispersion matrixes, respectively, *S*_*b*_ represents the distance from each class sample to the center of all class samples after projection, *S*_*w*_ represents the distance from each type of sample to the center of this type of sample after projection, and *W* represents the projection vector.

(12) $$\begin{array}{c} \left\{\begin{array}{c} S_{b}=\frac{1}{N}\sum_{i=1}^{c}n_{i}\left({\bar{x}}_{i}-\bar{x}\right){\left({\bar{x}}_{i}x_{ij}-\bar{x}\right)}^{T},\\ S_{w}=\frac{1}{N}\sum_{i=1}^{c}\sum_{j=1}^{n_{i}}n_{i}\left(x_{ij}-{\bar{x}}_{i}\right){\left(x_{ij}-{\bar{x}}_{i}\right)}^{T},\\ {\tilde{S}}_{b}=W^{T}S_{b}W,\\ {\tilde{S}}_{w}=W^{T}S_{w}W, \end{array}\right\}, \end{array}$$

where *N* is the total number of samples, *x*_*ij*_ represents the *j*th sample of the category *i*, and the number of samples in each type is *n*_*i*_, which is the mean value of the feature sample.

(13) $$\begin{array}{c} \left\{\begin{array}{c} {\bar{x}}_{i}=\frac{1}{n_{i}}\sum_{j=1}^{n_{i}}x_{ij}\\ \bar{x}=\frac{1}{C}\sum_{i=1}^{C}{\bar{x}}_{i} \end{array}\right\}, \end{array}$$

where ${\bar{x}}_{i}\;\left(i=1,2,\ldots ,C\right)$ is the mean of all data in the *i* type of samples, *n*_*i*_ data are available for each type of sample, and $\bar{x}$ is the mean of all types of sample data.

#### 2.2.3. Extreme Learning Machine (ELM)

ELM was proposed by Huang et al. [30] in 2004, which is a simple, easy-to-use, and effective single-hidden-layer feedforward neural network learning algorithm. The calculation process is presented as follows. The single-hidden-layer feedforward neural network can be expressed by the following equation:

(14) $$\begin{array}{c} \left\{\begin{array}{c} \sum_{i=1}^{L}\beta_{i}g\left(W_{i}\cdot X_{j}+b_{i}\right)=o_{j},\;j=1,2,\ldots ,N,\\ W_{i}={\left[w_{i1},w_{i2},\ldots ,w_{in}\right]}^{T}, \end{array}\right. \end{array}$$

where *g*(*x*) represents the activation function, *W*_*i*_ and *β*_*i*_, respectively, represent the input and output weights, *b*_*i*_ represents bias, and (*W*_*i*_ · *X*_*j*_) represents the inner product of *W*_*i*_ and *X*_*j*_. In a single-hidden-layer neural network, the goal of training is to achieve a minimum output error equal to zero, that is,

(15) $$\begin{array}{c} \sum_{j=1}^{N}\left\|o_{j}-t_{j}\right\|=0, \end{array}$$

where *β*_*i*_, *W*_*i*_, and *b*_*i*_ are available.

(16) $$\begin{array}{c} \left\{\begin{array}{c} \beta_{i}g\left(W_{i}\cdot X_{j}b_{i}\right)=t_{j},\;j=1,2,\ldots ,N,\\ H\beta =T, \end{array}\right. \end{array}$$

where the expression of the matrix in equation (16) is *Hβ*=*T*, *H* is the output of the hidden layer node, and *β* and *T*, respectively, denote the output weight and expected output.

(17) $$\begin{array}{c} H\left(W_{1},\ldots ,W_{l},b_{1},\ldots ,X_{1},\ldots ,X_{l}\right)\\ ={\left[\begin{array}{ccc} g\left(W_{1}\cdot X_{1}+b_{1}\right) & \cdots & g\left(W_{L}\cdot X_{1}+b_{L}\right)\\ \vdots & & \vdots \\ g\left(W_{1}\cdot X_{N}+b_{1}\right) & \cdots & g\left(W_{L}\cdot X_{N}+b_{L}\right) \end{array}\right]}_{N\times L},\\ \beta ={\left[\begin{array}{c} \beta_{1}^{T}\\ \vdots \\ \beta_{L}^{T} \end{array}\right]}_{L\times m},\\ T={\left[\begin{array}{c} T_{1}^{T}\\ \vdots \\ T_{N}^{T} \end{array}\right]}_{N\times m}, \end{array}$$

where *N* is the sample size of the training set, *L* is the number of hidden layer nodes, and *g*(*x*) is the activation function. Equation (18) is used to find $\hat{\beta_{i}}$, ${\hat{W}}_{i}$, and ${\hat{b}}_{i}$.

(18) $$\begin{array}{c} \left\|H\left({\hat{W}}_{i},{\hat{b}}_{i}\right){\hat{\beta }}_{i}-T\right\|=\underset{W,b,\beta }{\min}\left\|H\left(W_{i},b_{i}\right)\beta_{i}-T\right\|, \end{array}$$

where *i*=1,2,…, *L*. Equation (18) is equivalent to the minimum loss function.

(19) $$\begin{array}{c} E=\sum_{j=1}^{N}\left(\sum_{i=1}^{L}\beta_{i}g{\left(W_{i}\cdot X_{j}+b_{i}-t_{j}\right)}^{2}\right). \end{array}$$

The method of solving equation (19) is the training process. According to *Hβ*=*T*, if *H* is a square matrix, then the smallest error can be solved *β*=*H*^−1^*T*; if *H* is not a square, then *β*=*H*^+^*T*, where *H*^+^ is the Moore–Penrose generalized inverse of matrix *H*.

### 2.3. ABC-SVM Optimization Algorithm

ABC algorithm is widely presented to solve optimization problems due to its outstanding convergence characteristics [49, 50]. In this paper, the ABC algorithm is employed to optimize the kernel function parameter and penalty coefficients of the SVM to improve the accuracy and real-time performance of gait pattern recognition. A gait pattern recognition model is also established on the basis of the ABC-SVM optimization algorithm.

#### 2.3.1. ABC Algorithm

The ABC algorithm simulates the gathering honey process of bees and contains two parts: the food source and the colony. In this algorithm, each food source position corresponds to a possible solution of the problem optimization, and the amount of honey in the source corresponds to the fitness of each solution value [49, 50]. The bee colony is divided into three categories: employed bees, onlooker bees, and scout bees.

The search process of ABC is as follows. The employed bees determine a new food source in their search area based on the initial food source information. These bees then lead back to the hive and share the new food source information with the onlooker bees through dance, and the onlooker bees guide the bees based on the amount of honey. The returned information is optimized for the food source, and then the onlooker bees search for a new food source in the search area according to the food source information. When the food source is abandoned, the employed bees become scout bees and start to search for the food source randomly until the best food source location is found. The specific process is presented as follows.

Firstly, the parameters are initialized and set as follows, where NP is the size of the bee colony, FN = NP/2 is the number of food sources, LMT is the number of times of food termination cycle, and maxCycle is the maximum cycle coefficient. In addition, the food sources must be initialized according to equation (20), and the fitness value of each food is calculated.

(20) $$\begin{array}{c} x_{id}=x_{\min\cdot d}+\text{rand}\left(0,1\right)\left(x_{\max\cdot d}-x_{\min\cdot d}\right), \end{array}$$

where *x*_*id*_ is a *D* dimensional vector, *i*=1,2,…, (NP/2), and *d*=1,2,…, *D* is the number of parameters to be optimized.

Then, the search is started, the employed bees search the food source in the search area according to equation (21), and a new food source *V*_*id*_ is generated.

(21) $$\begin{array}{c} V_{id}=x_{id}+\varphi_{id}\left(x_{id}-x_{jd}\right), \end{array}$$

where *φ*_*id*_ indicates a random number between [−1,1], which is used to control the search area range, *j* ∈ [1, (NP/2)], and *d*=1,2,…, *D* is a randomly selected subscript and not equal to *i*.

The fit_*i*_ is calculated on the basis of the new food source information, and whether the food should be kept is determined on the basis of the greedy selection mechanism. The calculation formula is as follows:

(22) $$\begin{array}{c} {\text{fit}}_{i}=\left\{\begin{array}{cc} \frac{1}{\left(1+f_{i}\right)}, & f_{i}\geq 0,\\ 1+abs\left(f_{i}\right), & \text{other}, \end{array}\right. \end{array}$$

where *f*_*i*_ represents the objective function of the food source.

After completing the search task, the employed bees return to the hive and share the searched information of the food source with the onlooker bees. An onlooker bee selects a food source depending on the probability value associated with that food source; *p*_*i*_ is calculated by the following formula:

(23) $$\begin{array}{c} p_{i}=\frac{{\text{fit}}_{i}}{\sum_{i=1}^{\text{NP}/2}{\text{fit}}_{i}}, \end{array}$$

where fit_*i*_ is the fitness value of the solution *i* evaluated by its employed bees, which is proportional to the nectar amount of the food source in the position *i*. *p*_*i*_ is the selection probability of the current solution.

Subsequently, the onlooker bees will search in the selected food source field and determine the location of the new food source according to equation (21). Replacement of the old food source with a new food source is determined in accordance with the amount of food and through the greedy algorithm. If the number of times of the food source that has not been updated exceeds the limit time LMT, this food source is discarded. The corresponding employed bees are transformed into scout bees, and a new food source is randomly generated according to equation (20).

#### 2.3.2. ABC Algorithm for SVM Parameter Optimization (ABC-SVM)

**Figure 1:**
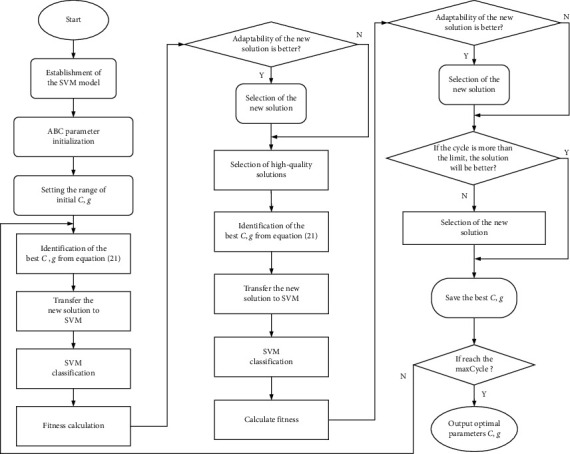
Flowchart of ABC-SVM optimization algorithm.

The ABC optimization algorithm is used in this study to optimize SVM parameters to solve the problem of SVM parameter selection. Compared with traditional parameter optimization algorithms, ABC has effective search capability and simple operations.  Figure 1 shows the ABC-SVM optimization algorithm flow, and the specific steps are as follows.

Firstly, the feature samples are normalized, and the SVM model is constructed. The ABC algorithm parameters are then initialized.

According to equations (21) and (22), the new optimal solution (parameters *g*, *C*, to be optimized) is determined, and the fitness value (classification accuracy in SVM) is solved. The food source with a high fitness value is maintained following the greedy selection method, and the onlooker bees select high-quality solutions according to equation (23).

The improvement of the solution is needed to be determined when the current number of cycles is larger than LMT, and the optimal solution information in the search process is saved. Otherwise, the solution is abandoned. Meanwhile, the employed bees become the scout bees, and a new solution is randomly generated following equation (20) to replace the abandoned old solution.

Finally, whether the number of cycles has reached maxCycle is determined. The optimal solution parameters *g* and *C* are outputted when reached; otherwise, the search starts again.

The selection of parameter values is a complex problem. If the colony size is too large, it will increase the iterative time of the algorithm, which is not conducive to the optimization search. LMT value controls the convergence of the algorithm and has an important influence on the algorithm to jump out of the local optimal solution. The parameter selection is based primarily on the previous experience [49, 51], and the parameters are determined by the data test results in time and space. The values of each specific event are different, but they all have a reasonable range, which can be verified by experiments.

According to the experimental experiences, the bee colony size is set to NP = 20, FN = 10, maxCycle = 30, and LMT = 300. The upper bound and lower bound of the SVM parameters are 100 and 0.01, respectively.

## 3. Results and Discussion

**Figure 2:**
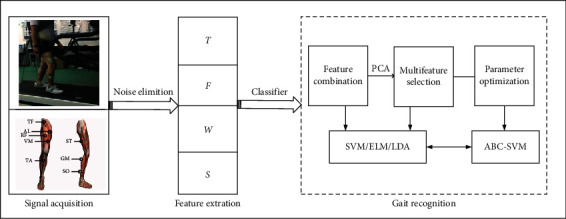
Schematical diagram of the gait recognition.

In this study, the experimental process and the algorithm flow consist of three parts: signal acquisition, feature extraction, and gait recognition. Firstly, sEMG data were supplied through experiments. Then, four types of features are extracted from the denoised sEMG signals, i.e., time-domain (*T*), frequency-domain (*F*), wavelet (*W*), and fuzzy entropy (*S*) features. Finally, these features are combined and selected and sent to various classifiers for gait recognition. The block diagram is given in Figure 2.

### 3.1. Experimental Data Collection

According to the function and contribution of muscles in different stages of normal gait and the sensitivity to sEMG collection equipment, eight muscles with similar characteristics to the signal source of sEMG signals are selected [3]. These muscles include the vastus medialis (VM), adductor longus (AL), tensor fascia lata (TF), semitendinosus (ST), rectus femoris (RF), tibialis anterior (TA), gastrocnemius (GM), and soleus (SO).

In the experiments, five healthy adult males aged 23–25 years old with heights of 168–178 cm were selected as subjects, and the subjects were required to refrain from exercising vigorously before the experiment. The EMG signal acquisition instrument (DataLINK SX230, sampling frequency: 1000 Hz) and the 3D motion capture system (Vicon, sampling frequency: 100 Hz) synchronously collected sEMG and 3D motion signals.

**Figure 3:**
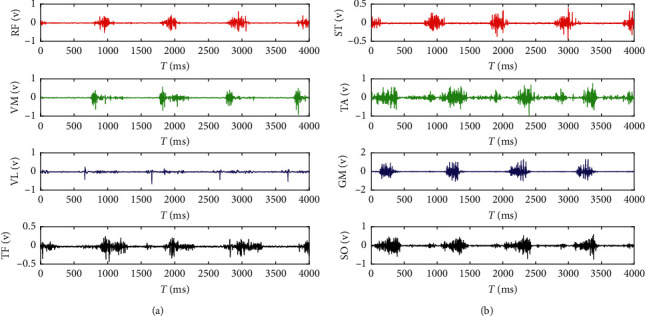
Collection of muscle sEMG signals. (a) First four muscles. (b) Rear four muscles.

At the beginning of the experiment, the experimenter walked at a constant speed on a treadmill at a speed of 1.37 m/s. Each group of experiments continued to walk 70 gaits at a constant speed. The collected sEMG signals are shown in Figure 3. The sEMG signals of each channel (different muscles) show differences, and the activation degree of the same muscle in the asynchronous state is different. Therefore, the sEMG signals can reflect the information of the asynchronous state.

### 3.2. sEMG Signal Data Processing

#### 3.2.1. Noise elimination

As a nonstationary weak bioelectric signal, the sEMG signals will be mixed with physiological noise and other interferences during the acquisition process. According to the noise characteristics of the acquisition system, the original sEMG signals are denoised by the wavelet transform modulus maximum method [47]. That is, the different change characteristics of the signal and noise modulus maxima on the wavelet scale are used to remove the noise in the signal.

#### 3.2.2. Feature Extraction

**Figure 4:**
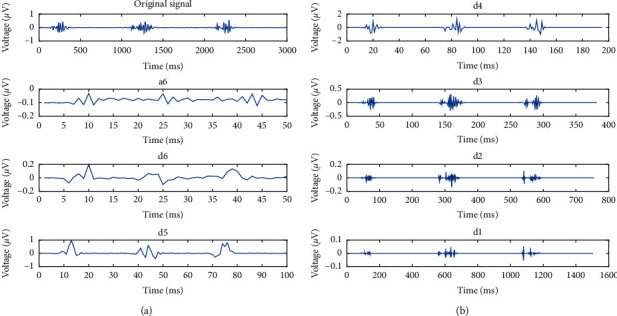
Wavelet feature extraction of semitendinosus signal.

Feature extraction is performed on the denoised data. According to the periodic continuous characteristics of the sEMG signals, the time-domain features, IAV, VAR, and ZC, are extracted via the translation window method [6]. The signal undergoes Fourier transform due to the unstable time-domain features to extract its MPF and MF as the frequency features [11]. Aiming at the nonstationary and nonlinear characteristics of the sEMG signals, as shown in Figure 4, the original sEMG signal is subjected to db5 wavelet six-layer decomposition, and the sixth layer decomposition is extracted to obtain the low-frequency approximate and high-frequency detail components [16]. The singular and mean values are calculated as wavelet features, and the fuzzy entropy of the sEMG signals is calculated as the nonlinear feature [18].

### 3.3. Recognition Results of Multiple Feature Combination

Walking gait is a periodic motion of alternating feet. This motion is divided into support and swing phases according to the different states of the soles of the feet touching and leaving the ground, which can then be further subdivided into multiple motion modes [3]. This study divides gait phases into prestance, midstance, terminal stance, and preswing. The subdivision of gait patterns will not be expanded, and the impact of different feature types and classification models on the recognition results will be discussed. Different feature combinations affect feature dimensionality reduction and ABC-SVM parameter optimization methods on the recognition results.

#### 3.3.1. Single Type of Feature Recognition with SVM

Firstly, four types of features, including time-domain (*T*), frequency-domain (*F*), wavelet (*W*), and fuzzy entropy (*S*) features, are inputted into the SVM for classification and recognition, respectively. The recognition results are shown in Table 1.

Table 1 shows that the four types of features are classified by SVM to achieve good recognition results. Meanwhile, the frequency-domain feature recognition effect is the best, with a recognition rate of 93.90%, followed by the time-domain feature sample recognition result with a recognition rate of 91.66%, the entropy feature sample with a recognition rate of 91.71%, and the wavelet feature with a recognition rate of 90.61%. In addition, the recognition rate of the frequency-domain and fuzzy entropy features in the midstance was significantly higher than other class feature samples, thereby supporting the recognition rate in the midstance. The classification results of the four types of features in the three other stages are similar.

#### 3.3.2. Single Type of Feature Recognition with LDA

Secondly, four types of features, including *T*, *F*, *W*, and *S*, are inputted into the LDA for classification and recognition. The recognition results are shown in Table 2.

Table 2 shows that the frequency-domain feature recognition rate in the LDA is the highest, thereby reaching 92.11%, followed by the FuEn feature recognition rate of 83.60% and the wavelet feature recognition rate of 80.86%. The time-domain feature is the last with a recognition rate of 80.06%. In addition, compared with the three other types of features, the frequency-domain features have a significantly high recognition rate in the midstance and terminal stance of gait support.

#### 3.3.3. Single Type of Feature Recognition with ELM

Thirdly, four types of features, i.e., *T*, *F*, *W*, and *S*, are inputted into the ELM for classification and recognition. The recognition results are shown in Table 3.

Table 3 shows that the recognition results of various types of feature samples are different after being inputted to the ELM. The recognition result of FuEn reaches 92.02%, followed by the time-domain feature samples with a recognition rate of 91.49%, the frequency-domain feature samples with a recognition rate of 87.30%, and finally the wavelet feature samples with a recognition rate of 82.87%.

Tables 1–3 reveal that the classification results of each type of feature in each period are different when the four types of features undergo gait pattern recognition through different classification models. This finding indicates that each type of feature is different during exercise. The sEMG signal information was analyzed from different directions.

#### 3.3.4. Comparison of Different Classifiers on Recognition Results

The four types of features, including *T*, *F*, *W*, and *S*, are inputted into SVM, LDA, and ELM for classification recognition. The recognition results are shown in Tables 4–7.

**Figure 5:**
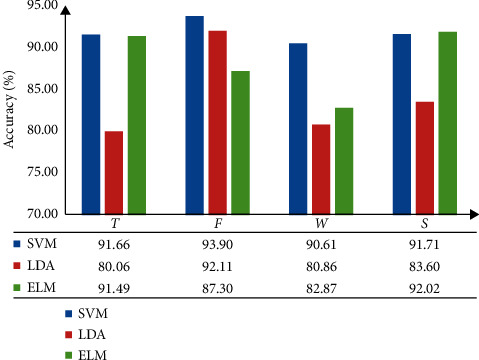
Comparison of classifier performance.

Tables 4–7 reveal that the classification effect is different when the feature samples are inputted to different classifiers. Tables 4–7 can be converted into histograms for analysis, and the results are shown in Figure 5.

Figure 5 shows the different recognition rates of the three types of classifiers for each feature sample. The classification performance of SVM and ELM is generally better than that of LDA. Meanwhile, the classification performance of SVM is stable. Therefore, follow-up investigations use SVM as the classification model.

#### 3.3.5. Feature Combination and Multiple Feature Selection

**Figure 6:**
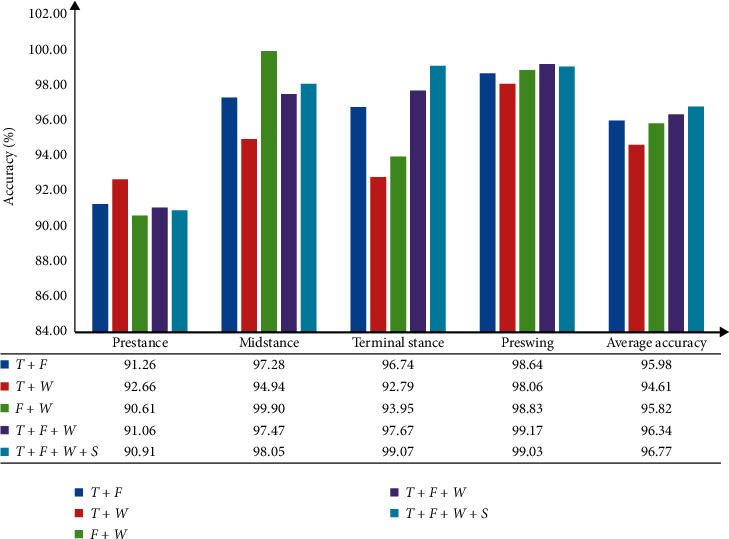
Classification results of multiple feature combination.

The analysis of Figure 5 indicates that different types of features have their characteristics. Therefore, this study finds the optimal combination sample by exploring different feature combinations and dimensionality reduction processing of feature combinations.  Figure 6 shows the recognition results of each feature combination.

Figure 6 shows that the recognition rate of combined features in time and frequency domains is 95.98%, that in time and wavelet domains is 94.61%, and that in frequency and wavelet domains is 95.82%. The feature recognition rate in time, frequency, and wavelet combination domains is 96.34%. Combining all features of this study to form the largest combined feature, the recognition rate is 96.77%.

In addition, with the continuous increase in feature types, the accuracy of gait pattern recognition in each period has been constantly improved. The classification performance of the classifier is the best when all features are involved in recognition, but the feature dimension is 80. The computational complexity will be considerably increased, which will lead to problems, such as extended training time. The principal component analysis (PCA) method is used in this study to reduce the dimension of the feature vector.  Table 8 shows the results of the PCA with multiple feature selection, in which feature numbers 1–10 represent MPF(*F*), FuEn(*S*), MF(*F*), the singular value of the detail component (*W*), IAV (*T*), ZC (*T*), VAR (*T*), the singular value of the approximate component (*W*), the mean of the detailed component (*W*), and the mean of the approximate component (*W*), respectively.

Table 8 shows that the principal components must be screened following the contribution rate to characterize the original feature information. A total of 95% of the original information was retained as the standard, and the first six principal components were selected to form the most excellent feature samples.  Table 9 shows the recognition results of multiple feature combination with SVM. Groups 1-2 represent all feature combinations and dimensionality reduction feature combination samples (optimum multiple feature selection/combination).

Table 9 reveals that the recognition result of all feature combinations is 96.77%, and that of the optimal feature combination is 96.48%. Compared with all feature combinations, the feature dimension of the optimal feature combination is high overall. After multiple feature selection by PCA optimization algorithm, the feature dimension is less than 32, which markedly reduces the training time.

### 3.4. Recognition Results of ABC-SVM Parameter Optimization

Firstly, the time-domain feature samples are inputted into the SVM and the ABC-SVM. Meanwhile, the optimized kernel function parameter is 23.1509, the penalty coefficient is 95.1204, and the recognition results are shown in Table 10.

Table 10 shows that the classification performance of the ABC algorithm-optimized SVM is better than the SVM, and the recognition rate is increased by 4.23% to 95.89%. Compared with other gait stages, the gait classification performance of the mid- and late-support periods has been significantly improved, thereby increasing by 8.97% and 5.35%, respectively.

The frequency-domain feature samples are inputted into the SVM model, and the ABC algorithm is used to optimize the SVM model. Meanwhile, the optimized kernel function parameter is 43.1927, the penalty coefficient is 70.9264, and the recognition results are shown in Table 11.

Table 11 shows that the classification performance of the ABC algorithm-optimized SVM is better than the SVM, and the recognition rate is increased by 2.73% to 96.63%.

The wavelet feature samples are inputted into the SVM model and the ABC algorithm to optimize the SVM model. Meanwhile, the optimized kernel function parameter is 4.3459, the penalty coefficient is 18.0331, and the recognition results are shown in Table 12.

Table 12 shows that compared with the SVM, the ABC algorithm optimizes the classification performance of the SVM, which increases the recognition rate by 3.86% to 94.47%. Compared with other gait stages, the gait supporting midstance classification performance has been significantly improved.

The FuEn feature samples are inputted into the SVM model and the ABC algorithm to optimize the SVM model. Meanwhile, the optimized kernel function parameter is 55.6746, the penalty coefficient is 19.6584, and the recognition results are shown in Table 13.

Table 13 reveals that the classification performance of the ABC algorithm-optimized SVM is better than the SVM, and the recognition rate is increased by 3.47% to 95.18%. Compared with other gait stages, the gait supporting midstance classification performance has been significantly improved.

The optimized feature samples after dimensionality reduction are inputted into the SVM model and the ABC algorithm to optimize the SVM model. Meanwhile, the optimized kernel function parameter is 4.4958, the penalty coefficient is 38.0182, and the recognition results are shown in Table 14.

Table 14 shows that the classification performance of the ABC algorithm-optimized SVM is better than the SVM, and the recognition rate is improved by 0.82% to 97.30% as presented in Tables 10–14. The recognition results of various and combined features before and after optimization are shown in Table 15. In Table 15, *H* represents the features of dimensionality reduction with PCA (results after optimization).

**Figure 7:**
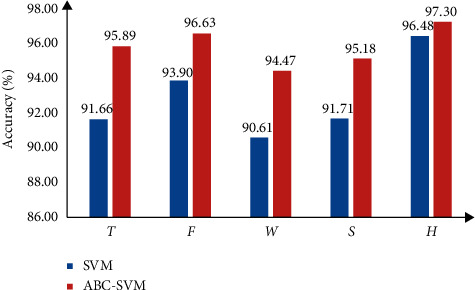
Feature recognition results.

Table 15 shows that each feature sample is recognized by the SVM and the ABC-SVM models. The feature recognition rate of various samples is improved.  Figure 7 shows the recognition of each feature sample after the optimization results.

Figure 7 shows that the classification results are significantly improved when various features of the sEMG signals are inputted through the ABC-optimized SVM classification model. This finding indicates that the ABC-SVM proposed in this paper can be used for gait phase recognition research to improve the classification performance of SVM effectively.

Using the existing pattern recognition method to recognize gait through sEMG signals, two problems should be addressed. First, because walking gait is a continuous periodic process, different stages of this continuous process are required for the classifier. Thus, this process should be divided correctly. Second, as a bioelectric signal generated by muscle contraction activity, sEMG contains a considerable amount of human motion and control information, from which the information required for gait recognition can be obtained. However, the sEMG signals cannot be fully decoded by the conventional feature extraction methods due to the sEMG nonlinear and nonstationary characteristics. Therefore, the performance of the classifier and the selection of feature samples are two key factors.

Considering the performance of the classifier, Figure 5 shows the comparison of different classifiers. The results are compared with LDA and ELM, and the classification performance of SVM is stable and accurate. The recognition rate in Table 15 shows that using ABC-SVM classifier has significantly better recognition performance than the SVM classifier, and the average recognition rate increased by 3.18%.When the time-domain, frequency-domain, wavelet, and FuEn features and combination feature samples are increased by 4.23%, 2.73%, 3.86%, 3.47%, and 0.82%, respectively, the ABC method can accurately and effectively find the optimal combination of SVM penalty coefficient and kernel function parameter. Therefore, the recognition error of ABC-SVM is reduced, and the recognition accuracy is increased. Using ABC to optimize SVM parameters has certain advantages in solving small sample and nonlinear pattern recognition problems.

In the selection of feature samples, Table 1 shows the recognition results of different features with SVM classifiers. This table also reveals that different feature samples have a considerable impact on the recognition results.  Figure 6 shows that with the continuous increase of types of features, the recognition effect is significantly better than other feature combinations, thereby reaching 96.77%. The PCA method is utilized to reduce the dimensionality of features and solve the problem of increasing training time due to the increase in feature dimensionality. Therefore, compared with other combinations, the PCA-based samples of multiple feature combinations proposed in this paper for the nonlinear and nonstationary characteristics of EMG signals have reduced the recognition errors.

## 4. Conclusions

The problem of human motion pattern recognition based on sEMG signals is studied in this paper. Considering classifier selection, SVM, LDA, and ELM are compared. Moreover, the classification accuracy and stability of SVM and ELM are better than LDA classifiers. The ABC algorithm is presented to optimize the parameters of the SVM classifier, and a higher recognition rate than the traditional SVM method is obtained. The sample selection of multiple feature combinations is based on the nonstationary and nonlinear characteristics of the sEMG signals. The statistical features of the signals are obtained via the time-domain analysis method, the frequency-domain features are extracted by the Fourier transform, the local and aperiodic features of the signal can be obtained through the wavelet transform, and the asynchronous state mode is obtained through the fuzzy entropy, respectively. Combining the four types of features and reducing the dimensionality, the recognition results of multiple feature combination samples are better than those of other feature samples. When combined with parameter optimization of ABC-SVM classifier, the recognition accuracy is significantly improved regardless of the single feature in ABC-SVM or the multiple feature combination with SVM, which can effectively improve the stability of overall gait recognition.

In this study, we propose a multiple feature combination of sample selection and ABC-SVM recognition algorithm, in gait recognition of normal subjects, which can not only increase the recognition accuracy but also improve the reliability of the recognition performance. If the experimental conditions permit, the proposed analysis method of gait recognition can also be extended to the case with walking dysfunction in the future.

## Figures and Tables

**Table 1:** Classification results of various features with SVM (%).

Sample	Prestance	Midstance	Terminal stance	Preswing
*T*	91.61	85.31	91.86	97.86
*F*	90.15	96.09	90.12	99.22
*W*	89.51	82.59	91.74	98.59
*S*	89.86	91.54	87.09	98.35

**Table 2:** Classification results of various features with LDA (%).

Sample	Prestance	Midstance	Terminal stance	Preswing
*T*	92.56	57.59	70.85	99.22
*F*	91.63	87.67	90.54	98.58
*W*	88.37	63.42	74.88	96.76
*S*	90.23	73.93	74.11	96.11

**Table 3:** Classification results of various features with ELM (%).

Sample	Prestance	Midstance	Terminal stance	Preswing
*T*	90.21	87.55	89.65	98.54
*F*	90.56	83.17	77.33	98.15
*W*	82.17	68.97	83.14	97.18
*S*	90.34	92.80	87.67	97.28

**Table 4:** Classification results of time-domain features with various classifiers (%).

Classifier	Prestance	Midstance	Terminal stance	Preswing
SVM	91.61	85.31	91.86	97.86
LDA	92.56	57.59	70.85	99.22
ELM	90.21	87.55	89.65	98.54

**Table 5:** Classification results of frequency-domain features with various classifiers (%).

Classifier	Prestance	Midstance	Terminal stance	Preswing
SVM	90.15	96.09	90.12	99.22
LDA	91.63	87.67	90.54	98.58
ELM	90.56	83.17	77.33	98.15

**Table 6:** Classification results of wavelet features with various classifiers (%).

Classifier	Prestance	Midstance	Terminal stance	Preswing
SVM	89.51	82.59	91.74	98.59
LDA	88.37	63.42	74.88	96.76
ELM	82.17	68.97	83.14	97.18

**Table 7:** Classification results of fuzzy entropy features with various classifiers (%).

Classifier	Prestance	Midstance	Terminal stance	Preswing
SVM	89.86	91.54	87.09	98.35
LDA	90.23	73.93	74.11	96.11
ELM	90.34	92.80	87.67	97.28

**Table 8:** Multiple feature analysis and selection with PCA.

Feature number	Eigenvalues	Contribution rate (%)
1	4.80	48.04
2	1.49	14.86
3	1.29	12.93
4	0.77	7.74
5	0.65	6.46
6	0.50	5.01
7	0.20	2.01
8	0.16	1.59
9	0.12	1.18
10	0.02	0.18

**Table 9:** Recognition results of multiple feature combination with SVM (%).

Group	Prestance	Midstance	Terminal stance	Preswing
1	90.91	98.05	99.07	99.03
2	91.07	97.67	98.26	98.93

**Table 10:** Results of two classifiers with time-domain feature (%).

Classifier	Prestance	Midstance	Terminal stance	Preswing
SVM	91.61	85.31	91.86	97.86
ABC-SVM	92.31	94.28	97.21	99.75

**Table 11:** Results of two classifiers with frequency-domain feature (%).

Classifier	Prestance	Midstance	Terminal stance	Preswing
SVM	90.15	96.09	90.12	99.22
ABC-SVM	92.66	97.96	96.86	99.03

**Table 12:** Results of two classifiers with wavelet feature (%).

Classifier	Prestance	Midstance	Terminal stance	Preswing
SVM	89.51	82.59	91.74	98.59
ABC-SVM	90.56	92.71	96.51	98.11

**Table 13:** Results of two classifiers with fuzzy entropy feature (%).

Classifier	Prestance	Midstance	Terminal stance	Preswing
SVM	89.86	91.54	87.09	98.35
ABC-SVM	90.21	96.98	93.72	99.81

**Table 14:** Results of two classifiers with optimal features (%).

Classifier	Prestance	Midstance	Terminal stance	Preswing
SVM	91.07	97.67	98.26	98.93
ABC-SVM	94.61	97.96	96.74	99.87

**Table 15:** Comparison of results before and after optimization (%).

Classifier	*T*	*F*	*W*	*S*	*H*
SVM	91.66	93.90	90.61	91.71	96.48
ABC-SVM	95.89	96.63	94.47	95.18	97.30

## Data Availability

The data used to support the findings of this study are available from the corresponding author upon request.
